# Primary enteric-type adenocarcinoma of the renal parenchyma in a patient with bilateral renal malrotation: a case report and review of the literature

**DOI:** 10.3389/fonc.2026.1977843

**Published:** 2026-10-14

**Authors:** Yuanqi Wu, Haoke Zheng, Gaurab Pokhrel, Rongyu Zhao, Shouchang Wu, Shuanbao Yu, Xuepei Zhang

**Affiliations:** Department of Urology, The First Affiliated Hospital of Zhengzhou University, Zhengzhou, China

**Keywords:** case report, primary enteric-type adenocarcinoma, renal malrotation, renal parenchyma, robotic-assisted partial nephrectomy

## Abstract

**Background:**

Primary enteric-type adenocarcinoma of the renal parenchyma is an exceptionally rare malignancy, accounting for less than 1% of renal epithelial tumors. Due to its morphological and immunohistochemical overlap with metastatic gastrointestinal malignancy, establishing a primary renal origin presents a considerable diagnostic challenge. Radical nephroureterectomy has traditionally been the standard surgical treatment.

**Case presentation:**

We report a 47-year-old asymptomatic female with an incidental 68 mm cystic-solid left renal mass detected during a trauma evaluation. Imaging suggested Bosniak category IV on computed tomography and cystic teratoma on magnetic resonance imaging. Notably, the patient had bilateral renal malrotation. Serum tumor markers (CEA and CA19-9) were within normal limits. The patient underwent successful robotic-assisted partial nephrectomy with a warm ischemia time of 15 minutes. Histopathology revealed adenocarcinoma with an enteric immunophenotype: positive for CK20, CDX-2, SATB2, and Villin, with complete loss of PAX-8 and GATA-3. Postoperative gastrointestinal endoscopy and ^18^F-FDG PET-CT excluded primary gastrointestinal or distant occult malignancies, confirming a primary renal parenchymal origin. At one-month follow-up, the patient showed no evidence of recurrence without adjuvant therapy.

**Conclusion:**

This case represents the first reported association of primary enteric-type renal adenocarcinoma with bilateral renal malrotation and demonstrates that robotic-assisted partial nephrectomy can be a feasible nephron-sparing approach for selected patients with this aggressive tumor. A systematic diagnostic approach including surgical resection, immunohistochemical profiling, and exclusion of gastrointestinal metastasis is essential for accurate diagnosis and optimal management.

## Introduction

Primary enteric-type adenocarcinoma of the renal parenchyma is an extraordinarily rare malignancy accounting for less than 1% of renal epithelial tumors, distinguished from the typical renal pelvic variant by its parenchymal localization and elusive histogenesis. Radiologically and pathologically, these lesions pose formidable diagnostic challenges; they frequently mimic Bosniak III/IV cystic renal masses while exhibiting extreme lineage plasticity—characterized by robust enteric differentiation (CDX-2, CK20, SATB2, and Villin positivity) coupled with the complete loss of canonical renal and urothelial markers (PAX-8 and GATA-3). Because this immunophenotypic signature is virtually indistinguishable from metastatic colorectal adenocarcinoma, confirming a genuine renal primary demands a rigorous exclusionary workup incorporating gastrointestinal endoscopy and functional whole-body PET-CT imaging. Furthermore, while radical nephroureterectomy has historically served as the default surgical paradigm, congenital anomalies such as bilateral renal malrotation may impair gravitational drainage and induce chronic microenvironmental stasis predisposing to metaplastic transformation, highlighting the clinical necessity of nephron-sparing strategies to preserve long-term renal function in young patients. Herein, we describe the first documented case of primary renal parenchymal enteric-type adenocarcinoma in a 47-year-old female with congenital bilateral renal malrotation, successfully treated via robotic-assisted partial nephrectomy (RAPN), alongside a systematic 10-year literature review evaluating diagnostic algorithms, lineage reprogramming, and the feasibility of nephron-sparing surgery.

## Case presentation

A 47-year-old female was referred to our department following the incidental detection of a left renal mass during a trauma evaluation 10 days prior. The patient was asymptomatic, with no relevant physical findings. Her medical history was significant only for a previously resected ovarian serous papillary cystadenoma. She reported no personal or family history of gastrointestinal malignancies, polyps, inflammatory bowel disease, or hereditary tumor syndromes. Routine laboratory investigations, including serum carcinoembryonic antigen (CEA) and carbohydrate antigen 19-9 (CA19-9), were within normal limits.

Contrast-enhanced abdominal CT revealed a well-circumscribed, mixed cystic-solid mass in the lower pole of the left kidney, measuring 55 mm × 54 mm × 69 mm (23 HU, 39 HU, and 60 HU). The solid component (18 mm × 16 mm) exhibited marked enhancement, consistent with a Bosniak category IV lesion. The mass abutted and compressed the left ureter, causing secondary hydronephrosis. Bilateral renal malrotation with anteriorly oriented hila was also noted. Following contrast-enhanced computed tomography which confirmed a Bosniak category IV lesion, complementary multiparametric magnetic resonance imaging (MRI) was deliberately performed for three key indications. First, MRI provided superior soft-tissue contrast resolution to differentiate dense proteinaceous or hemorrhagic fluid from subtle enhancing mural nodules and micro-septa. Second, diffusion-weighted imaging (DWI) and apparent diffusion coefficient (ADC) mapping quantitatively evaluated tumor cellularity, distinguishing complex cystic teratoma from high-grade malignancy. Finally, multiplanar MRI precisely delineated the parenchymal resection margin relative to the ventrally rotated hilar vessels and compressed ureter, providing an indispensable surgical roadmap. Abdominal magnetic resonance imaging (MRI) showed bilateral renal malrotation with a heterogeneous cystic-solid lesion measuring 53 mm × 57 mm × 68 mm, with short-T1 and long-T2 signal intensities and hyperintensity on high b-value diffusion-weighted imaging (**Figure 1**). Dynamic contrast-enhanced MRI demonstrated mild heterogeneous enhancement of the solid component with no cystic enhancement. A preoperative diagnosis of cystic teratoma was considered.

**Figure 1:**
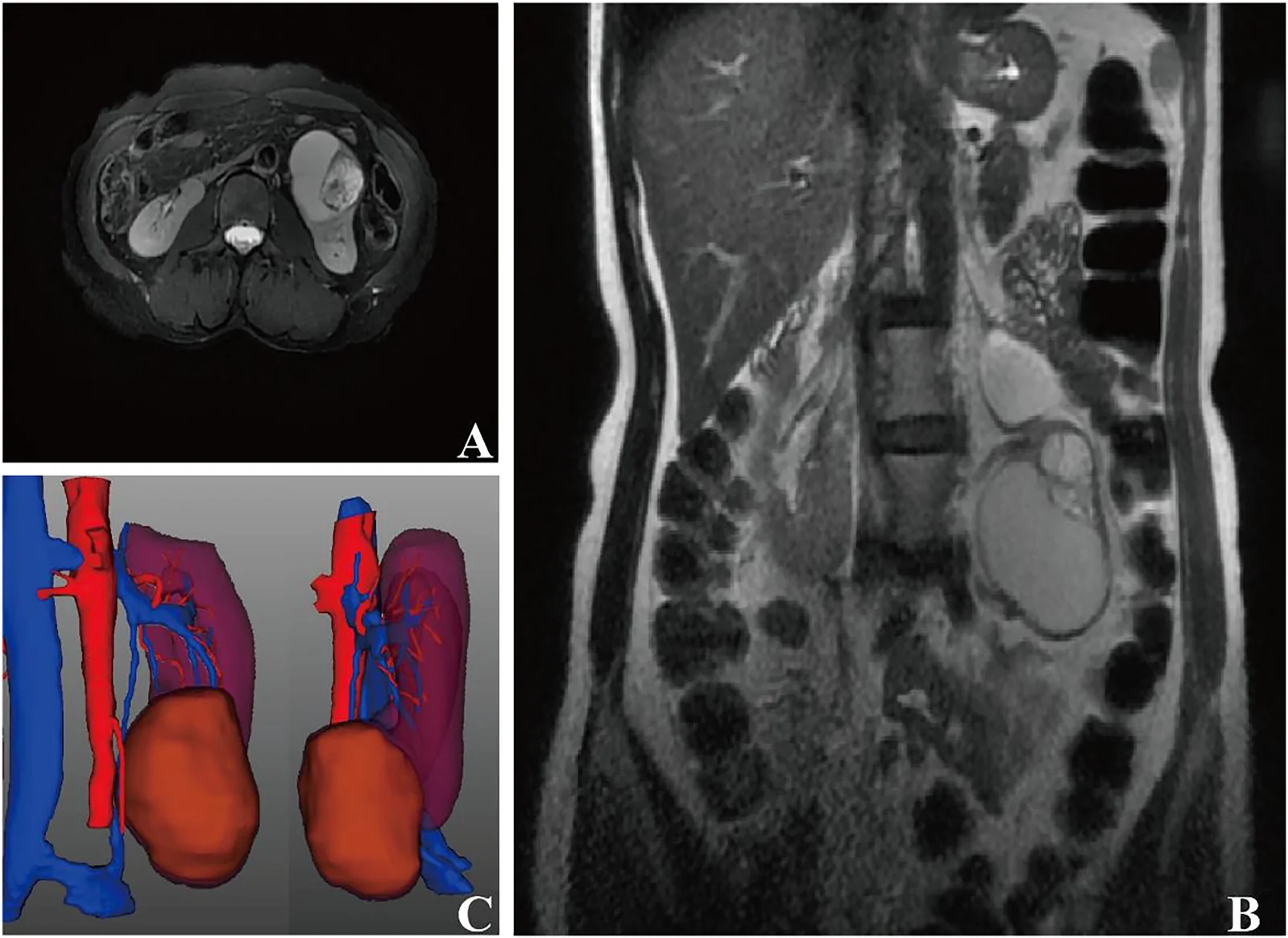
Preoperative imaging evaluations of the patient with primary renal parenchymal enteric-type adenocarcinoma. **(A)** Axial view demonstrating a mass-like abnormal signal in the anterior aspect of the lower pole of the left kidney, measuring approximately 53 mm×57 mm×68 mm; **(B)** Coronal MRI view illustrating a sharply circumscribed, mixed cystic-solid lesion in the lower pole of the left kidney; **(C)** Three-dimensional (3D) digital reconstruction model depicting the spatial anatomical relationship between the orange-coded tumor in the anterior-lower pole of the left renal parenchyma and major abdominal vasculature (abdominal aorta in red; inferior vena cava in blue), with preserved functional renal parenchyma clearly visualized.

The patient underwent robotic-assisted partial nephrectomy. The clinical decision to pursue robotic-assisted partial nephrectomy (RAPN) over radical nephroureterectomy was justified by several imperative considerations. In a 47-year-old patient harboring congenital bilateral renal malrotation, maximal nephron preservation was essential to prevent long-term renal functional decline and secondary stasis-related complications. Anatomically, preoperative imaging confirmed an exophytic lower-pole tumor with distinct cleavage planes from the main renal vasculature, ensuring R0 resectability. Moreover, because imaging could not exclude indolent cystic teratoma, radical nephrectomy posed significant overtreatment risks. Intraoperatively, a mass approximately 6.0 cm × 7.0 cm was seen at the lower pole of the left kidney, adherent to the ipsilateral gonadal vessels. The tumor was carefully dissected and complete excision was done. Total operative time was 84 minutes, with a warm ischemia time of 15 minutes. Gross pathological examination of the specimen (6.2 cm × 5.8 cm × 4.4 cm) demonstrated a nodular, cystic-solid tumor measuring 5.4 cm × 5.0 cm × 3.5 cm with a smooth, grayish-white cyst wall and gelatinous, grayish-yellow solid areas. The resection margin was negative. Microscopy examination showed diffusely distributed atypical glands lined by pleomorphic columnar epithelial cells with frequent mitoses, intraluminal mucin secretion, and stromal lymphocytic infiltration (**Figure 2**). Immunohistochemistry demonstrated positivity for AE1/AE3, CK8/18, CK7, CK20, CDX-2, SATB2, and Villin. PAX-8 and GATA-3 were completely negative, while estrogen receptor and progesterone receptor were negative and Her-2 showed 1+ expression. The Ki-67 proliferation index was markedly elevated at approximately 70%.

**Figure 2:**
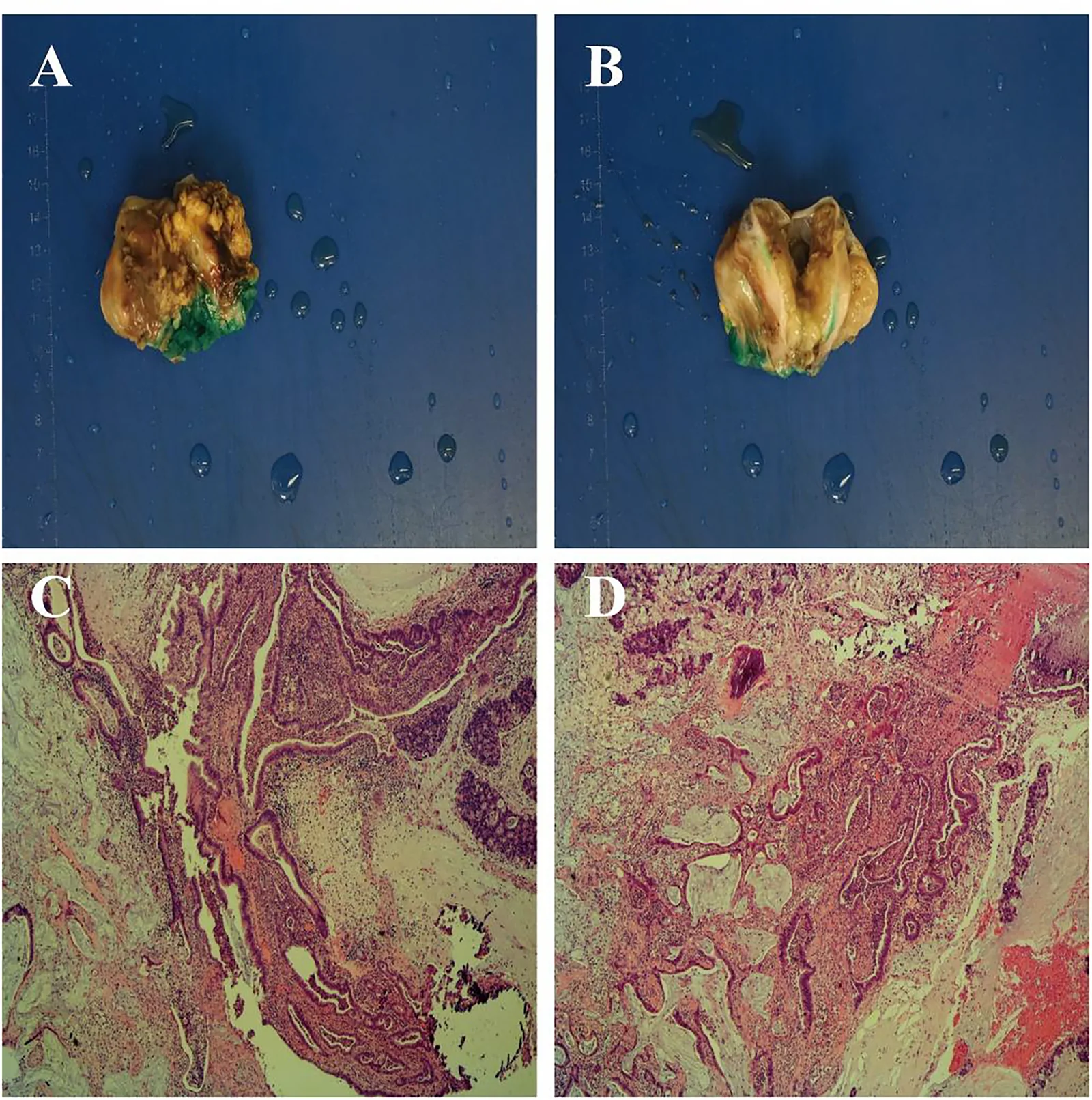
Gross and microscopic pathological findings of the primary renal parenchymal enteric-type adenocarcinoma. **(A, B)** Gross pathological inspection of the resected left renal mass 6.2 cm×5.8 cm×4.4 cm. A minor amount of perinephric fat is attached to the capsular side, which is easily separable from the capsule. The parenchymal resection margin surface measures 4.8 cm×3.0 cm, with a minimum distance of 0.4 cm to the surgical margin. Sectioning demonstrates a nodular, cystic-solid tumor measuring 5.4 cm×5.0 cm×3.5 cm. The inner cyst wall appears smooth and grayish-white (thickness: ~0.1 cm). The solid cut surface is grayish-yellow with moderate consistency, exhibiting a gelatinous/myxoid texture. Histological morphology and immunophenotype are consistent with adenocarcinoma showing enteric differentiation. **(C, D)** Microscopic examination demonstrating diffusely distributed atypical glands lined by pleomorphic columnar epithelial cells with frequent mitotic figures. Mucin secretion within glandular lumens, intratumoral and stromal lymphocytic infiltration, and complete loss of normal renal parenchymal architecture are prominently observed(×20).

To exclude an occult gastrointestinal primary, comprehensive postoperative evaluation was performed. Total gastrointestinal endoscopy revealed no mucosal abnormalities. ^18^F-FDG PET-CT demonstrated no hypermetabolic foci outside the expected postoperative inflammatory changes at the left renal surgical site. The absence of gastrointestinal symptoms, negative tumor markers, and unremarkable endoscopic and PET-CT findings, a metastatic gastrointestinal origin was excluded, establishing the final diagnosis of primary renal parenchymal enteric-type adenocarcinoma. At one-month follow-up, the patient was in good health condition with no evidence of recurrence or metastasis.

## Literature review methodology

A systematic literature search was conducted in the PubMed database for articles published between January 2016 and August 2026 to identify all documented cases of primary renal mucinous or enteric-type adenocarcinoma. Eligibility criteria required peer-reviewed, English-language publications reporting histopathologically confirmed primary renal neoplasms with complete patient-level clinicopathological, surgical, and follow-up data. Exclusion criteria comprised secondary metastases from extra-renal malignancies, urothelial carcinomas exhibiting focal glandular differentiation, and conference abstracts lacking primary individual data. Overall, 14 publications detailing 15 discrete cases met all criteria and were synthesized into **Table 1**.

**Table 1:** Summary of primary renal mucinous adenocarcinoma cases reported in the literature over the past decade.

Author & year	Patient details & clinical presentation	Tumor characteristics & histopathology	Management & clinical outcome
Xie et al. (2026) (11)	79-year-old male presenting with left abdominal pain and a long-standing history of nephrolithiasis; endoscopic evaluation identified a mass in the left renal pelvis.	Histopathological examination confirmed high-grade tubular adenocarcinoma of the left renal pelvis.	Laparoscopic left radical nephroureterectomy; uneventful postoperative recovery; currently under regular surveillance with no evidence of recurrence.
Gupta et al. (2026) (12)	75-year-old male presenting with a 6-month history of persistent left flank pain; imaging demonstrated left renal calculi accompanied by left hydronephrosis.	Histopathology revealed primary mucinous adenocarcinoma of the kidney, exhibiting glandular and villous architecture with neoplastic glands invading the subepithelial connective tissue.	Radical nephrectomy followed by adjuvant systemic chemotherapy with gemcitabine and cisplatin; currently under regular postoperative surveillance.
Huynh et al. (2026) (13)	55-year-old male presenting with bilateral flank pain and fever; imaging demonstrated right renal and ureteral calculi accompanied by severe hydronephrosis.	Histopathology confirmed moderately differentiated mucinous adenocarcinoma; the tumor was composed of atypical gland-forming cells featuring hyperchromatic nuclei, moderate pleomorphism, and moderate mitotic activity, arranged in clusters and elongated structures with focal glandular lumina formation.	Right nephrectomy; currently under regular postoperative surveillance.
Taylor et al. (2025) (14)	60-year-old female presenting with persistent right flank pain, a history of long-standing nephrolithiasis, recurrent urinary tract infections (UTIs), and chronic renal insufficiency; imaging revealed a mass in the lower pole of the right kidney, preoperatively suspected as xanthogranulomatous pyelonephritis (XGP).	Histopathology confirmed XGP with an incidental finding of primary mucinous adenocarcinoma *in situ* of the renal pelvis; intestinal metaplastic changes were identified at the mucosal margins.	Right radical nephrectomy; developed local recurrence postoperatively (invading the flank soft tissue and adjacent ribs), subsequently underwent resection of the recurrent mass followed by 6 months of adjuvant mFOLFOX6 chemotherapy; currently under regular follow-up.
Gazzah et al. (2024) (15)	34-year-old male presenting with a several-month history of persistent back pain and no hematuria; imaging demonstrated a mass in the right renal pelvis exhibiting marked enhancement following iodinated contrast administration.	Histopathology confirmed primary enteric-type adenocarcinoma of the renal pelvis.	Right nephroureterectomy; currently under regular surveillance with no evidence of recurrence.
Nueva España-Pérez & Pedroza (2024) (16)	55-year-old male presenting with left abdominal/flank pain, hematuria, and pyuria; imaging demonstrated multiple renal cysts accompanied by hydronephrosis and nephrolithiasis.	Histopathology revealed markedly invasive mucinous adenocarcinoma with signet-ring cell features; extensive glandular metaplasia extending from the proximal ureter up to the renal pelvis, lined by intestinal-type columnar epithelium with goblet cells admixed with malignant glands.	Radical nephrectomy; currently under regular postoperative surveillance.
Gali et al. (2024) (17) (Case 1)	50-year-old male presenting with left flank pain, passage of mucinous/gelatinous discharge in urine, and elevated serum creatinine; imaging demonstrated severe hydronephrosis restricted to the upper pole of the left kidney.	Histopathology revealed a mucinous borderline cystic tumor with invasive microcarcinoma; the renal pelvic system was markedly dilated and filled with mucin, accompanied by parenchymal thinning and tiny papillary projections.	Left upper-pole partial nephrectomy; regular postoperative follow-up; developed tumor recurrence and distant metastasis 1 year postoperatively.
Gali et al. (2024) (17) (Case 2)	53-year-old male with a horseshoe kidney presenting with right abdominal pain and a palpable mass, with a history of laparoscopic cyst decortication for a right renal cyst at an outside institution 6 months prior (histopathology *post-hoc* revealed mucinous adenocarcinoma); imaging demonstrated a well-defined, multilocular, non-enhancing hypodense cystic lesion in the right subcostal region extending to the right lumbar area.	Histopathology confirmed mucinous adenocarcinoma; multiple cystic spaces replaced the renal parenchyma, filled with gelatinous material and scattered extravasated mucin pools.	Radical nephrectomy; currently under regular postoperative surveillance with no evidence of recurrence.
Zeng et al. (2023) (18)	55-year-old male presenting without subjective discomfort (asymptomatic); imaging demonstrated a large cystic hypodense lesion in the left kidney with partial cyst wall calcification.	Histopathology confirmed mucinous adenocarcinoma, revealing abundant extracellular mucin within the fibrous cyst wall, with tumor cells distributed sporadically or arranged in glandular architectures.	Laparoscopic radical nephrectomy; currently under regular surveillance with no evidence of recurrence.
Li et al. (2023) (19)	64-year-old male presenting without subjective discomfort (asymptomatic, incidentally detected during health examination); imaging demonstrated a renal cystic mass, preoperatively suspected/misdiagnosed as a cystic teratoma.	Histopathology confirmed mucinous adenocarcinoma; gross examination revealed gelatinous/colloidal tumor tissue accompanied by calcified structures.	Nephron-sparing surgery (NSS/partial nephrectomy) followed by adjuvant gemcitabine and cisplatin chemotherapy; regular postoperative follow-up; developed distant metastasis and disease recurrence 8 years postoperatively.
Zou et al. (2023) (20)	64-year-old female presenting with a >1-year history of right lower back pain; imaging demonstrated right renal enlargement, multiple hyperdense nodules within the right kidney, and wall thickening of the right ureter.	Histopathology confirmed a high-grade mucinous neoplasm; gross and microscopic examination revealed dilation of the renal pelvis, marked renal parenchymal thinning, and abundant pools of gelatinous mucin partially deposited within the stroma of the cyst wall.	Laparoscopic right nephrectomy; currently under regular postoperative surveillance with no evidence of recurrence.
Han et al. (2021) (21)	42-year-old male presenting with a >5-year history of intermittent left abdominal pain; imaging demonstrated a mixed-density mass in the left kidney with filling defects within the left renal vein and adjacent inferior vena cava (IVC).	Histopathology confirmed primary mucinous adenocarcinoma of the left renal pelvis with tumor invasion into the left renal vein and IVC; neoplastic cells were oval-to-columnar with abundant pale cytoplasm and hyperchromatic round-to-polygonal nuclei, arranged in irregular glandular ducts or ridge-like structures within extracellular mucin pools.	Left radical nephrectomy combined with inferior vena cava resection and artificial vascular graft replacement; currently under regular postoperative surveillance with no evidence of recurrence.
Li et al. (2020) (22)	66-year-old male presenting with a 2-month history of fever and right flank pain; imaging demonstrated renal pelvic calculi, ureteral stricture, and severe hydronephrosis accompanied by marked cortical thinning.	Histopathological examination revealed primary mucinous adenocarcinoma of the renal pelvis.	Open radical nephrectomy; uneventful postoperative recovery; currently under regular follow-up with no evidence of recurrence.
Gupta et al. (2020) (23)	31-year-old male presenting with a 1-year history of right flank discomfort; imaging demonstrated a hyperdense mass in the right renal pelvis accompanied by right hydronephrosis.	Histopathology revealed primary adenocarcinoma *in situ* of the renal pelvis and ureter, featuring neoplastic cells arranged in glandular patterns floating within extracellular mucin pools, with invasion extending into the superficial mucosa, lamina propria, and muscularis layer of the renal pelvis.	Right nephrectomy; currently under regular postoperative surveillance with no evidence of recurrence.
Lai & Teng (2016) (9)	40-year-old male presenting with a 1-year history of intermittent right flank pain; imaging demonstrated a cystic-solid mass in the right kidney with enhancing mural nodules.	Histopathology confirmed primary enteric-type adenocarcinoma of the renal pelvis (intestinal-type tubular adenocarcinoma) with abundant pools of extravasated mucin, primarily confined to the renal pelvis with infiltration into the adjacent renal parenchyma.	Right radical nephrectomy; empirically treated with postoperative Interleukin-2 (IL-2) and ubenimex to prevent recurrence; currently under regular surveillance with no evidence of recurrence.

## Discussion

Primary enteric-type adenocarcinoma of the kidney is an exceptionally rare malignancy, with fewer than 60 cases reported to date, all of which have originated from the renal pelvis (**Table 1**). To our knowledge, a primary enteric-type adenocarcinoma localized within the renal parenchyma not involving the renal pelvic, has not been previously documented (1). In this case, the tumor presented as an asymptomatic incidentaloma that closely mimicked cystic renal cell carcinoma (RCC) on multimodal imaging, posing a significant diagnostic challenge. Immunohistochemically, the tumor exhibited complete loss of canonical renal lineage markers (PAX-8 and GATA-3) and strong expression of colorectal associated markers (CDX2 and CK20). This immunophenotypic combination creates a diagnostic dilemma, as a primary renal neoplasm with an enteric profile is indistinguishable from a metastatic colorectal adenocarcinoma. Addressing this diagnostic difficulty requires a rigorous exclusionary clinical workup, combined with careful consideration of the molecular mechanisms that may underlie unusual pathways of renal oncogenesis. Our case presents several distinctive features that merit detailed discussion.

Histopathology and immunohistochemistry are essential for definitive diagnosis of renal masses. PAX-8 is the most sensitive and specific marker for confirming renal lineage, with a positivity rate exceeding 90% in renal cell carcinomas (2). Similarly, GATA-3, ltia marker for urothelial and mammary carcinomas, is frequently positive in primary renal pelvic adenocarcinomas. The absence of PAX-8 is particularly significant, as it definitively excludes a primary renal cell carcinoma, while GATA-3 negativity rules out urothelial (transitional cell) origin. This immunophenotype, combined with negative gastrointestinal endoscopy and PET-CT, firmly establishes the diagnosis of a primary renal parenchymal tumor rather than a metastatic gastrointestinal adenocarcinoma. This case demonstrates that PAX-8 negativity in a renal adenocarcinoma should not be interpreted as definitive evidence of metastasis, but rather necessitates a systematic, multimodal investigative approach.

The complete loss of PAX-8 expression in this case suggests substantial phenotypic reprogramming or lineage plasticity during tumor development. Although PAX-8 is a lineage defining transcription factor for renal parenchymal cells, certain tumors undergoing extensive enteric metaplasia may entirely silence their organ specific markers (3). The high expression of CDX-2 and CK20 indicates that the transcriptional program was co-opted by an enteric differentiation pathway (4). Analogous lineage switching has been documented in primary enteric-type adenocarcinomas of extra-intestinal sites, such as the lung and ovary (5). We propose that this tumor represents an extreme lineage divergence of renal epithelial cells during malignant transformation, questioning the conventional view that PAX-8 negativity definitively excludes a primary renal malignancy (6).

Regarding the histogenesis of PEAK, two primary mechanisms have been proposed. The first is the metaplasia-dysplasia-carcinoma sequence, wherein chronic inflammation or nephrolithiasis induces enteric metaplasia of the urothelium, ultimately leading to malignant transformation (7). The second is the multipotent stem cell hypothesis, which suggests that dormant embryonic stem cell remnants within the renal parenchyma undergo aberrant differentiation along an enteric lineage triggered by oncogenic signals (8).

The metaplasia-dysplasia-carcinoma sequence provides a possible framework for understanding the association with renal malrotation in this case. Bilateral renal malrotation, a congenital anomaly resulting in impaired urinary drainage, may predispose to chronic stasis, infection, and inflammation, conditions conducive to urothelial metaplasia. To our knowledge, this is the first reported case of PEAK in a patient with renal malrotation. While this association is not definitive, it raises the possibility that congenital anomalies predisposing to urinary stasis may represent an unrecognized risk factor warranting further investigation.

Standardized management guidelines for PEAK are currently lacking. Historically, radical nephrectomy has been the preferred approach for aggressive or rare renal malignancies (9, 10). In this case, however, robotic-assisted partial nephrectomy was successfully performed with negative margins (0.4 cm) and a warm ischemia time of 15 minutes. This outcome suggests that nephron-sparing surgery may be appropriate for selected patients with localized, early-stage tumors, provided complete excision is achieved. This case supports individualized surgical decision-making based on tumor characteristics and anatomical feasibility, rather than a uniform radical approach.

The prognosis for primary enteric-type renal adenocarcinoma is poor. A review of the literature reports a 2-year overall survival of approximately 50% following surgical resection, indicating the aggressive nature of these tumors (7). Poor prognostic factors include high-grade histology, advanced pathological stage, and positive surgical margins. Our patient’s tumor exhibited high-grade features and a Ki-67 proliferation index of approximately 70%, both of which are concerning. However, the negative surgical margin and absence of metastatic disease at presentation are favorable. Given the potential for late recurrence, we recommend contrast-enhanced CT or MRI of the chest, abdomen, and pelvis every 3–6 months for the first two years, followed by annual imaging for up to five to ten years. Serum tumor markers are unreliable for surveillance. The role of adjuvant therapy has not been established; our patient was managed with observation alone. In cases of systemic progression, emerging molecular evidence suggests that PEAK shares genomic alterations with colorectal adenocarcinoma (e.g., KRAS, APC, TP53), providing a rationale for considering gastrointestinal-directed cytotoxic regimens over standard RCC-targeted therapies (4).

## Conclusion

Primary enteric-type adenocarcinoma of the renal parenchyma is a rare and aggressive tumor that presents significant diagnostic challenges. This case highlights the importance of a systematic approach comprising surgical resection, comprehensive immunohistochemical profiling, and rigorous exclusion of gastrointestinal primaries. We report the first association of this tumor with bilateral renal malrotation and demonstrate that robotic-assisted partial nephrectomy may be feasible in selected patients. Clinicians should recognize that renal tumors can undergo phenotypic changes that result in the loss of their characteristic immunohistochemical markers. Multidisciplinary collaboration among urologists, pathologists, radiologists, and medical oncologists is essential for optimal management. Further molecular characterization of this malignancy will guide future diagnostic and therapeutic strategies.

## Data Availability

The original contributions presented in the study are included in the article/supplementary material. Further inquiries can be directed to the corresponding author.
